# Supplementary material for: Environmental sustainability assessment of biodiesel production from Jatropha curcas L. seeds oil in Pakistan
Source: PLoS One. 2021 Nov 18;16(11):e0258409. doi: 10.1371/journal.pone.0258409 (PMC8601503; doi:10.1371/journal.pone.0258409)
Supplement: S1 Table — (DOCX) [file pone.0258409.s001.docx]

**Supporting Information**

**Table A1:** Emissions to water from cultivation of *JC* plantation during 2019-2020 in Pakistan.

| **Substance** |  | **Unit** | **Total** |
| --- | --- | --- | --- |
| 1-Pentene |  | µg | 9.056781 |
| 2,4-D amines |  | µg | 4.549756 |
| 2,4-D ester |  | µg | 1.001112 |
| Acetaldehyde |  | µg | 803.1477 |
| Acetic acid |  | mg | 3.387619 |
| Acetone |  | µg | 53.15618 |
| Acetyl chloride |  | µg | 9.414866 |
| Acidity, unspecified |  | mg | 5.264731 |
| Acids, unspecified |  | pg | 10.22766 |
| Aluminium |  | g | 27.02032 |
| Aluminium hydroxide |  | ng | 54.33355 |
| Ammonia |  | mg | 75.12605 |
| Cadmium |  | mg | 22.18816 |
| Calcium |  | g | 191.6866 |
| Carbon |  | µg | 116.0659 |
| Carbon disulfide |  | µg | 51.05127 |
| Carbonate |  | mg | 615.3839 |
| Chloride |  | g | 783.0875 |
| Chlorides, unspecified |  | mg | 151.5009 |
| Chlorine |  | mg | 26.50571 |
| Chloroacetyl chloride |  | µg | 14.57503 |
| Cobalt |  | mg | 164.8304 |
| Copper |  | g | 1.182613 |
| Ethanol |  | mg | 499.8181 |
| Ethene |  | mg | 1.834788 |
| Hydrogen chloride |  | mg | 14.75071 |
| Hydroxide |  | µg | 347.9035 |
| Iron |  | g | 22.28512 |
| Lead |  | mg | 232.8139 |
| Magnesium |  | g | 81.17959 |
| Mercury |  | mg | 2.429426 |
| Methane |  | pg | 37.8464 |
| Methanol |  | mg | 5.559079 |
| Nitric acid |  | pg | 310.906 |
| Nitrogen |  | mg | 29.74199 |
| Nitrogen dioxide |  | pg | 0.000519 |
| Nitrogen, atmospheric |  | mg | 129.4525 |
| Oxygen |  | ng | 5.532662 |
| Petroleum oil |  | mg | 29.37247 |
| Potassium |  | g | 54.60067 |
| Silicon |  | g | 155.9884 |
| Silver |  | mg | 2.552536 |
| Sodium |  | g | 497.0792 |
| Sodium chlorate |  | µg | 24.52942 |
| Sulfur |  | mg | 688.313 |
| Uranium-235 |  | mBq | 286.8258 |
| Urea |  | µg | 8.761459 |
| Zinc |  | g | 1.586022 |
