# Supplementary material for: Environmental sustainability assessment of biodiesel production from Jatropha curcas L. seeds oil in Pakistan
Source: PLoS One. 2021 Nov 18;16(11):e0258409. doi: 10.1371/journal.pone.0258409 (PMC8601503; doi:10.1371/journal.pone.0258409)
Supplement: S2 Table — (DOCX) [file pone.0258409.s002.docx]

**Supporting Information**

**Table A2:** Emissions to soil from cultivation of *JC* plantation in Pakistan during 2019-2020.

| **Substance** |  | **Unit** | **Total** |
| --- | --- | --- | --- |
| Aluminium |  | mg | 562.9486 |
| Ammonia |  | mg | 324.1752 |
| Arsenic |  | mg | 1.103294 |
| Arsenic V |  | pg | 7.011058 |
| Barium |  | mg | 272.5309 |
| Bromine |  | µg | 8.599726 |
| Cadmium |  | µg | 551.3044 |
| Calcium |  | g | 23.8948 |
| Carbon |  | g | 1.75016 |
| Carbon dioxide, to soil or biomass stock |  | g | 10.17697 |
| Chloride |  | g | 2.262863 |
| Chlorine |  | µg | 223.8073 |
| Choline chloride |  | µg | 90.66832 |
| Chromium |  | mg | 11.14681 |
| Chromium III |  | µg | 1.05559 |
| Chromium VI |  | mg | 2.442329 |
| Cobalt |  | µg | 39.07658 |
| Copper |  | mg | 42.57003 |
| Curium alpha |  | mBq | 243.0946 |
| Decane |  | mg | 1.957601 |
| Fluoride |  | mg | 30.94475 |
| Fungicides, unspecified |  | mg | 1.520921 |
| Heat, waste |  | J | 2.609973 |
| Herbicides, unspecified |  | mg | 1.133827 |
| Hydrocarbons, unspecified |  | µg | 166.9923 |
| Insecticides, unspecified |  | µg | 92.1022 |
| Iodine-129 |  | µBq | 3.820344 |
| Iron |  | g | 1.277916 |
| Kaolin |  | µg | 68.37825 |
| Lead |  | mg | 1.387529 |
| Magnesium |  | g | 4.003278 |
| Manganese |  | mg | 35.3044 |
| Mercury |  | µg | 13.20911 |
| Nickel |  | mg | -0.74875 |
| Nitrate |  | µg | 646.3148 |
| Nitrogen |  | pg | 329.2344 |
| Nitrogen, atmospheric |  | µg | 716.2314 |
| Organic carbon |  | µg | 274.5046 |
| Potassium |  | g | 2.915878 |
| Silicon |  | mg | 111.9536 |
| Silver |  | ng | 43.35597 |
| Sodium |  | g | 30.73769 |
| Sulfate |  | mg | 16.22335 |
| Sulfide |  | mg | 58.34994 |
| Sulfur |  | mg | 375.3015 |
| Sulfuric acid |  | mg | 7.21336 |
| Uranium-235 |  | mBq | 3.474014 |
| Uranium-238 |  | mBq | 53.87328 |
| Zinc |  | mg | 20.60176 |
