# Supplementary material for: Environmental sustainability assessment of biodiesel production from Jatropha curcas L. seeds oil in Pakistan
Source: PLoS One. 2021 Nov 18;16(11):e0258409. doi: 10.1371/journal.pone.0258409 (PMC8601503; doi:10.1371/journal.pone.0258409)
Supplement: S3 Table — (DOCX) [file pone.0258409.s003.docx]

**Supporting Information**

**Table A3:** Emissions to Air from cultivation of *JC* plantation in Pakistan during 2019-2020

| **Substance** |  | **Unit** | **Total** |
| --- | --- | --- | --- |
| Acetic acid |  | mg | 112.4515 |
| Acetone |  | mg | 32.61591 |
| Aluminium |  | mg | 688.8413 |
| Americium-241 |  | nBq | 0.003349 |
| Amine oxide |  | ng | 52.59309 |
| Ammonia |  | g | 7.223539 |
| Ammonium carbonate |  | µg | 64.70382 |
| Ammonium chloride |  | ng | 18.07218 |
| Ammonium, ion |  | ng | 0.184173 |
| Cadmium |  | mg | 1.375639 |
| Calcium |  | mg | 60.3512 |
| Carbon |  | µg | 33.91839 |
| Carbon dioxide |  | kg | 5.302339 |
| Carbon dioxide, biogenic |  | g | 592.7265 |
| Carbon dioxide, fossil |  | kg | 20.18311 |
| Carbon dioxide, land transformation |  | g | 477.4965 |
| Carbon monoxide |  | g | 4.233086 |
| Carbon monoxide, biogenic |  | g | 10.38495 |
| Carbon monoxide, fossil |  | g | 32.48952 |
| Carbon monoxide, land transformation |  | g | 4.274636 |
| Chloride |  | mg | 4.71647 |
| Chlorine |  | mg | 21.35849 |
| Chloroform |  | µg | 237.0367 |
| Chromium |  | mg | 11.11445 |
| Cobalt |  | mg | 1.611635 |
| Cobalt-60 |  | mBq | 1.691337 |
| Copper |  | mg | 11.15404 |
| Ethane |  | g | 1.623381 |
| Ethanol |  | mg | 48.69693 |
| Ethene |  | mg | 209.5586 |
| Fluoride |  | mg | 2.98478 |
| Fluorine |  | mg | 1.933683 |
| Formic acid |  | mg | 45.21748 |
| Heat, waste |  | MJ | 29.04928 |
| Helium |  | mg | 35.64454 |
| Hydrogen |  | g | 1.70802 |
| Hydrogen peroxide |  | µg | 2.866623 |
| Hydrogen sulfide |  | mg | 288.8818 |
| Iodine |  | mg | 9.661871 |
| Iron |  | mg | 81.05555 |
| Lead |  | mg | 10.38981 |
| Magnesium |  | mg | 36.43265 |
| Manganese |  | mg | 4.623845 |
| Mercury |  | µg | 706.3098 |
| Nitrogen dioxide |  | g | 9.990912 |
| Nitrogen fluoride |  | pg | 177.7282 |
| Nitrogen monoxide |  | µg | 110.7761 |
| Nitrogen oxides |  | g | 63.92438 |
| Nitrogen, atmospheric |  | g | 4.230547 |
| Organic carbon |  | µg | 84.3609 |
| Oxygen |  | g | 8.076297 |
| Ozone |  | mg | 69.78804 |
| Silver |  | µg | 6.575639 |
| Sodium |  | mg | 33.41833 |
| Sodium chlorate |  | µg | 33.28018 |
| Sulfur |  | ng | 12.87867 |
| Uranium-235 |  | mBq | 310.0785 |
| Water |  | kg | 9.742226 |
| Zinc |  | mg | 17.04286 |
