# Supplementary material for: Environmental sustainability assessment of biodiesel production from Jatropha curcas L. seeds oil in Pakistan
Source: PLoS One. 2021 Nov 18;16(11):e0258409. doi: 10.1371/journal.pone.0258409 (PMC8601503; doi:10.1371/journal.pone.0258409)
Supplement: S4 Table — (DOCX) [file pone.0258409.s004.docx]

**Supporting Information**

**Table A4:** Emissions to water from JC oil extraction phase in Pakistan during 2019-2020.

| **Substance** |  | **Unit** | **Total** |
| --- | --- | --- | --- |
| 1-Butanol |  | mg | 8.633 |
| 1-Pentanol |  | µg | 340.548 |
| Aluminium |  | kg | 1.097 |
| Ammonia |  | mg | 33.160 |
| Antimony |  | g | 1.657 |
| Barium |  | g | 35.944 |
| Benzene |  | g | 1.0520 |
| Beryllium |  | mg | 756.932 |
| BOD5, Biological Oxygen Demand |  | kg | 1.754 |
| Boron |  | g | 89.324 |
| Bromine |  | g | 37.1058 |
| Cadmium |  | g | 2.697 |
| Calcium |  | kg | 11.644 |
| Carbon |  | mg | 4.555 |
| Carbonate |  | g | 2.572 |
| Chloride |  | kg | 111.865 |
| Chlorine |  | mg | 61.570 |
| Chloroform |  | µg | 441.392 |
| Chromium |  | g | 3.002 |
| Cobalt |  | g | 10.646 |
| COD, Chemical Oxygen Demand |  | kg | 2.362 |
| Copper |  | g | 38.169 |
| Cyanide |  | mg | 431.143 |
| Fluoride |  | g | 200.250 |
| Formic acid |  | µg | 180.806 |
| Hydrogen chloride |  | mg | 750.752 |
| Iodide |  | mg | 840.001 |
| Iron |  | kg | 1.222 |
| Lead |  | g | 4.931 |
| Magnesium |  | kg | 5.394 |
| Mercury |  | mg | 582.264 |
| Nickel |  | g | 29.013 |
| Nitrate |  | kg | 23.558 |
| Nitrogen, atmospheric |  | g | 19.902 |
| Phosphorus |  | g | 532.610 |
| Potassium |  | kg | 3.6059 |
| Silicon |  | kg | 6.467 |
| Silver |  | mg | 433.46 |
| Sodium |  | kg | 64.70 |
| Tin |  | g | 1.9453 |
| Urea |  | µg | 232.389 |
| Zinc |  | g | 129.418 |
