# Supplementary material for: Environmental sustainability assessment of biodiesel production from Jatropha curcas L. seeds oil in Pakistan
Source: PLoS One. 2021 Nov 18;16(11):e0258409. doi: 10.1371/journal.pone.0258409 (PMC8601503; doi:10.1371/journal.pone.0258409)
Supplement: S5 Table — (DOCX) [file pone.0258409.s005.docx]

**Supporting Information**

**Table A5:** Emissions to soil from JC oil extraction phase in Pakistan during 2019-2020.

| **Substance** |  | **Unit** | **Total** |
| --- | --- | --- | --- |
| Aluminium |  | g | 4.437 |
| Antimony |  | µg | 227.566 |
| Barium |  | g | 1.956 |
| Boron |  | mg | 50.257 |
| Bromine |  | mg | 2.241 |
| Cadmium |  | g | 8.120 |
| Calcium |  | g | 21.555 |
| Carbon |  | g | 12.970 |
| Chloride |  | g | 15.369 |
| Chlorine |  | mg | 58.323 |
| Chromium |  | g | 94.394 |
| Cobalt |  | mg | 1.899 |
| Copper |  | g | 13.776 |
| Fluoride |  | mg | 234.648 |
| Iron |  | g | 26.721 |
| Lead |  | g | 7.952 |
| Magnesium |  | g | 3.772 |
| Mercury |  | µg | 353.623 |
| Mineral oil |  | g | 1.173 |
| Nickel |  | g | 15.362 |
| Nitrate |  | mg | 168.425 |
| Nitrogen, atmospheric |  | mg | 3.959 |
| Oils, biogenic |  | mg | 678.226 |
| Phosphorus |  | mg | 396.154 |
| Potassium |  | g | 2.504 |
| Silicon |  | g | 2.3460 |
| Silver |  | µg | 11.258 |
| Sodium |  | g | 8.978 |
| Sulfur |  | g | 3.831 |
| Sulfuric acid |  | mg | 207.431 |
| Tin |  | µg | 41.187 |
| Zinc |  | g | 97.964 |
