# Supplementary material for: Environmental sustainability assessment of biodiesel production from Jatropha curcas L. seeds oil in Pakistan
Source: PLoS One. 2021 Nov 18;16(11):e0258409. doi: 10.1371/journal.pone.0258409 (PMC8601503; doi:10.1371/journal.pone.0258409)
Supplement: S6 Table — (DOCX) [file pone.0258409.s006.docx]

**Supporting Information**

**Table A6:** Emissions to air from JC oil extraction phase in Pakistan during 2019-2020.

| **Substance** |  | **Unit** | **Total** |
| --- | --- | --- | --- |
| 1-Butanol |  | µg | 69.945 |
| 1-Pentanol |  | µg | 141.894 |
| Aluminium |  | g | 40.811 |
| Ammonia |  | kg | 18.094 |
| Antimony |  | g | 2.002 |
| Barium |  | mg | 912.726 |
| Benzene |  | g | 11.1636 |
| Beryllium |  | mg | 1.0730 |
| Boron |  | g | 1.613 |
| Bromine |  | mg | 591.70 |
| Cadmium |  | mg | 184.930 |
| Calcium |  | g | 2.217 |
| Carbon |  | mg | 1.331 |
| Carbon monoxide |  | mg | 1.110 |
| Chloride |  | ng | 6.448 |
| Chlorine |  | g | 1.2365 |
| Chloroform |  | mg | 10.954 |
| Chromium |  | g | 1.909 |
| Cobalt |  | mg | 178.709 |
| Copper |  | g | 2.215 |
| Cyanide |  | g | 3.189 |
| Fluoride |  | mg | 2.244 |
| Formic acid |  | mg | 986.367 |
| Helium |  | mg | 271.563 |
| Hydrogen |  | g | 4.0763 |
| Hydrogen chloride |  | g | 68.473 |
| Iodine |  | mg | 304.108 |
| Iron |  | g | 6.773 |
| Lead |  | g | 1.504 |
| Magnesium |  | g | 1.634 |
| Mercury |  | mg | 26.631 |
| Nickel |  | g | 2.2031 |
| Nitrate |  | mg | 94.440 |
| Nitrogen, atmospheric |  | g | 36.946 |
| Phosphorus |  | mg | 140.818 |
| Potassium |  | g | 2.774 |
| Silicon |  | g | 6.660 |
| Silver |  | µg | 215.347 |
| Sodium |  | g | 2.125 |
| Sulfuric acid |  | mg | 142.23 |
| Tin |  | mg | 193.394 |
| Zinc |  | g | 5.3510 |
