# Supplementary material for: Environmental sustainability assessment of biodiesel production from Jatropha curcas L. seeds oil in Pakistan
Source: PLoS One. 2021 Nov 18;16(11):e0258409. doi: 10.1371/journal.pone.0258409 (PMC8601503; doi:10.1371/journal.pone.0258409)
Supplement: S7 Table — (DOCX) [file pone.0258409.s007.docx]

**Supporting Information**

**Table A7:** Emissions to water from JC oil conversion phase in Pakistan during 2019-2020.

| **Substance** |  | **Unit** | **Total** |
| --- | --- | --- | --- |
| 1-Butanol |  | mg | 38.978 |
| 1-Pentanol |  | mg | 5.503 |
| Acetic acid |  | g | 2.483 |
| Acetone |  | mg | 76.379 |
| Aluminium |  | kg | 1.569 |
| Ammonia |  | mg | 54.314 |
| Antimony |  | mg | 724.449 |
| Arsenic |  | g | 3.502 |
| Barium |  | g | 32.377 |
| Benzene |  | g | 3.354 |
| Beryllium |  | mg | 905.444 |
| Boron |  | g | 30.072 |
| Bromine |  | g | 18.785 |
| Cadmium |  | mg | 755.289 |
| Calcium |  | kg | 11.447 |
| Carbon |  | µg | 768.801 |
| Chloride |  | kg | 28.616 |
| Chlorine |  | mg | 23.632 |
| Chloroform |  | mg | 6.667 |
| Chromium |  | g | 1.755 |
| Cobalt |  | g | 11.446 |
| Copper |  | g | 37.580 |
| Cyanide |  | mg | 125.343 |
| Ethanol |  | g | 2.162 |
| Fluorene |  | mg | 2.182 |
| Fluoride |  | g | 48.652 |
| Formic acid |  | mg | 2.9220 |
| Heat, waste |  | MJ | 7.327 |
| Iodide |  | g | 5.534 |
| Iron |  | kg | 1.390 |
| Lead |  | g | 1.5197 |
| Lithium |  | g | 80.473 |
| Mercury |  | mg | 169.601 |
| Nickel |  | g | 47.307 |
| Nitrate |  | kg | 31.931 |
| Nitrogen, atmospheric |  | g | 4.654 |
| Oxygen |  | ng | 255.832 |
| Paraffins |  | mg | 2.449 |
| Phosphorus |  | g | 157.142 |
| Potassium |  | kg | 3.756 |
| Silicon |  | kg | 10.783 |
| Silver |  | mg | 194.477 |
| Sodium |  | kg | 19.076 |
| Tin |  | mg | 586.480 |
| Vanadium |  | g | 7.329 |
| VOC, volatile organic compounds, unspecified origin |  | g | 1.1488 |
| Zinc |  | g | 73.198 |
