# Supplementary material for: Environmental sustainability assessment of biodiesel production from Jatropha curcas L. seeds oil in Pakistan
Source: PLoS One. 2021 Nov 18;16(11):e0258409. doi: 10.1371/journal.pone.0258409 (PMC8601503; doi:10.1371/journal.pone.0258409)
Supplement: S8 Table — (DOCX) [file pone.0258409.s008.docx]

**Supporting Information**

**Table A8:** Emissions to soil from JC oil conversion phase in Pakistan during 2019-2020.

| **Substance** |  | **Unit** | **Total** |
| --- | --- | --- | --- |
| Acetamide |  | mg | 2.088 |
| Aluminium |  | g | 3.136 |
| Antimony |  | µg | 83.533 |
| Arsenic |  | mg | 1.628 |
| Barium |  | g | 1.264 |
| Boron |  | mg | 47.757 |
| Bromine |  | µg | 828.121 |
| Cadmium |  | mg | 227.463 |
| Calcium |  | g | 18.034 |
| Carbon |  | g | 10.101 |
| Chloride |  | g | 9.327 |
| Chlorine |  | mg | 21.552 |
| Chromium |  | mg | 796.987 |
| Cobalt |  | mg | 1.0726 |
| Copper |  | g | -7.678 |
| Fluoride |  | mg | 211.889 |
| Insecticides, unspecified |  | µg | 197.806 |
| Iron |  | g | 15.187 |
| Kaolin |  | µg | 147.133 |
| Lead |  | g | 1.006 |
| Lithium |  | µg | 54.008 |
| Magnesium |  | g | 2.908 |
| Mercury |  | mg | 8.056 |
| Nickel |  | g | -2.4625 |
| Nitrate |  | mg | 62.238 |
| Nitrogen, atmospheric |  | mg | 1.772 |
| Phosphorus |  | mg | 397.663 |
| Potassium |  | g | 2.400 |
| Silicon |  | g | 2.647 |
| Silver |  | µg | 4.158 |
| Sodium |  | g | 5.346 |
| Tin |  | µg | 24.1771 |
| Vanadium |  | mg | 1.134 |
| Zinc |  | g | -18.625 |
