# Supplementary material for: Environmental sustainability assessment of biodiesel production from Jatropha curcas L. seeds oil in Pakistan
Source: PLoS One. 2021 Nov 18;16(11):e0258409. doi: 10.1371/journal.pone.0258409 (PMC8601503; doi:10.1371/journal.pone.0258409)
Supplement: S9 Table — (DOCX) [file pone.0258409.s009.docx]

**Supporting Information**

**Table A9:** Emissions to air from JC oil conversion phase in Pakistan during 2019-2020

| **Substance** |  | **Unit** | **Total** |
| --- | --- | --- | --- |
| 1-Butanol |  | mg | 13.216 |
| 1-Pentanol |  | mg | 2.293 |
| Acetamide |  | mg | 48.258 |
| Acetic acid |  | g | 480.647 |
| Acetone |  | g | 141.749 |
| Aluminium |  | g | 49.568 |
| Ammonia |  | g | 646.516 |
| Antimony |  | mg | 737.372 |
| Arsenic |  | mg | 145.983 |
| Barium |  | mg | 589.857 |
| Benzene |  | g | 100.397 |
| Beryllium |  | mg | 1.37 |
| Boron |  | g | 3.756 |
| Bromine |  | g | 1.291 |
| Cadmium |  | mg | 37.264 |
| Calcium |  | g | 2.388 |
| Carbon |  | µg | 224.666 |
| Carbon monoxide |  | mg | 1.818 |
| Chloride |  | ng | 10.562 |
| Chlorine |  | g | 2.647 |
| Chloroform |  | mg | 66.642 |
| Chromium |  | mg | 553.650 |
| Cobalt |  | mg | 54.257 |
| Copper |  | mg | 742.758 |
| Cyanide |  | g | 775.758 |
| Ethanol |  | g | 4.485 |
| Fluorene |  | ng | 731.830 |
| Fluoride |  | mg | 3.676 |
| Formic acid |  | g | 250.929 |
| Heat, waste |  | MJ | 25.948 |
| Helium |  | mg | 141.077 |
| Iodine |  | mg | 669.927 |
| Iron |  | g | 4.816 |
| Lead |  | mg | 547.254 |
| Lithium |  | ng | 38.374 |
| Mercury |  | mg | 24.732 |
| Nickel |  | mg | 587.734 |
| Nitrate |  | mg | 50.573 |
| Nitrogen, atmospheric |  | g | 49.367 |
| Ozone |  | g | 4.900 |
| Paraffins |  | µg | 550.874 |
| Phosphorus |  | mg | 99.992 |
| Potassium |  | g | 3.844 |
| Silicon |  | g | 5.7313 |
| Silver |  | µg | 296.465 |
| Sodium |  | mg | 843.598 |
| Tin |  | mg | 64.9700 |
| Uranium |  | µg | 701.330 |
| Vanadium |  | mg | 812.666 |
| VOC, volatile organic compounds |  | mg | 916.856 |
| Zinc |  | mg | 981.725 |
