# Supplementary figures and images for: Environmental sustainability assessment of biodiesel production from Jatropha curcas L. seeds oil in Pakistan
Source: PLoS One. 2021 Nov 18;16(11):e0258409. doi: 10.1371/journal.pone.0258409 (PMC8601503; doi:10.1371/journal.pone.0258409)

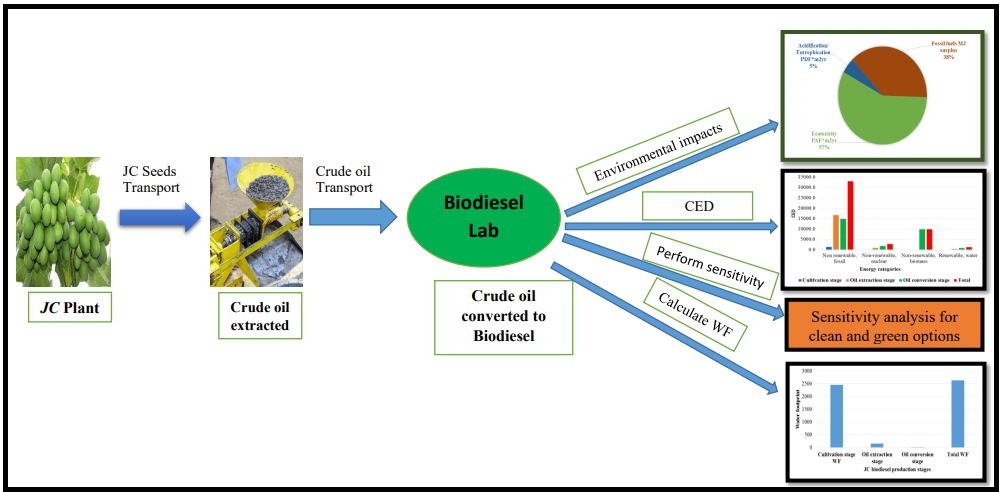

Supplement: S1 Graphical abstract — (PNG) [file pone.0258409.s010.PNG]
