# Supplementary material for: Environmental sustainability assessment of biodiesel production from Jatropha curcas L. seeds oil in Pakistan
Source: PLoS One. 2021 Nov 18;16(11):e0258409. doi: 10.1371/journal.pone.0258409 (PMC8601503; doi:10.1371/journal.pone.0258409)
Supplement: Attachment — Submitted filename: Reviewers Comments (PLOS-D-21-22104).docx [file pone.0258409.s011.docx]

The manuscript “Environmental sustainability assessment of biodiesel production from *Jatropha curcas L.* seeds oil in Pakistan” provide the insight on the environmental impacts of the commercial scale JC biodiesel production. The paper is interesting and provide valuable information. This paper is “**recommended for the publication**” in the PLOS journal. However, **few modifications** are recommended prior to publication

- Careful revision of manuscript for formatting is recommended.
- It is recommended to provide the status of *JC* biodiesel production in Pakistan, as it would be useful for readers to understand the importance of this study from Pakistani perspective.
- In future assumption section, point c mention that soybean oil process data was consider for the analysis instead of *JC* oil data due to software limitations. In my opinion it would off set the parameters. Please justify this point.
- Please to page 7, line 175, the mentioned reference is not according to the recommended style.
- Please clarify the statement at page 9 from line 231-235, as in present form it is ambiguous.
- In manuscript it is recommended that 20% reduction in baseline fossil fuel, synthetic fertilizers, and electricity use provide significant results. It would be valuable if the impact of this condition on the *JC* biodiesel production is also mentioned.
- It is recommended to delete table 5 as this information was also provided in the text and this table does not provide any other significant information.
